# Global regulatory trends of genome editing technology in agriculture and food

**DOI:** 10.1270/jsbbs.23046

**Published:** 2024-02-22

**Authors:** Masashi Tachikawa, Makiko Matsuo

**Affiliations:** 1 Graduate School of Environmental Studies, Nagoya University, Furo-cho, Chikusa, Nagoya, Aichi 464-8601, Japan; 2 Graduate School of Public Policy, The University of Tokyo, 7-3-1 Hongo, Bunkyo, Tokyo 113-0033, Japan

**Keywords:** agriculture, food, genome editing technology, regulation

## Abstract

There is a need to introduce new regulations regarding genome editing technology and its application to agriculture and food. Regulations are different among countries and sometimes inconsistent. Here, we summarize the current regulations regarding the use of genome editing technology in agriculture and food in various countries around the world. We also discuss the main regulatory developments expected to occur in the future.

## Introduction

The present paper provides an overview of the regulations that have been introduced and are being considered in Japan and abroad concerning the application of genome editing technology. Indeed, the regulations for plants generated using genome editing technology differ from country to country and can be inconsistent (Menz *et al.* 2020, Turnbull *et al.* 2021). These discrepancies reflect the existing differences in the regulations for genetically modified organisms (GMOs) among countries, and international harmonization is considered extremely difficult. It is necessary to examine how these differences will affect future innovation and trade. Here, we provide an overview of the current regulatory trends in each country regarding genome editing in the field of agriculture and food. We also discuss the main regulatory developments in the United States of America, the European Union, South America, Oceania, and Asia, including Japan.

The regulations in each country at this time may be classified into four types, as shown in Table 1. The classification is based on the following two points. First, whether or not products produced by genome editing technologies are considered GMOs, and second, what type of regulation is applied (whether or not GMO regulations are applied as is, and whether or not confirmation by the regulatory authority is required). Although this classification is tentative, it may be useful in classifying the regulatory patterns of various countries. Specific regulatory details for above-mentioned countries are described below in turn. The information in this paper is based on the regulatory status as of July 2023.

## Regulations in the United States of America

In the United States, the Department of Agriculture (USDA), Food and Drug Administration (FDA), and Environmental Protection Agency (EPA) have been regulating genetically modified organisms (GMOs) since 1986 based on the Coordinated Framework. However, there has been discussions about updating biotechnology regulations, resulting in calls to revise regulations. Under these circumstances, the USDA issued an official notice on May 18, 2020, to significantly revise its biotech regulations, i.e., 7 CFR 340. The new regulation was called the SECURE rule when it was published in May 2020. The EPA also published a final revision of the Federal Insecticide, Fungicide, and Rodenticide Act (FIFRA) regarding Plant-incorporated protectants (PIPs) derived from genome editing in May 2023. Additionally, the FDA collected comments in January 2017 regarding food derived from new plant varieties and initiated a discussion with the USDA regarding genome-edited animals in January 2021. Below is a summary of the regulatory policy of each agency.

### USDA regulatory policy

The USDA has been regulating transgenic crops based on the authority of the Plant Protection Act, which concerns plant pest risk. As mentioned above, the USDA made a major revision of the Code of Federal Regulation, 7 CFR 340, in May 2020. In this revised CFR, the USDA addressed the regulation of organisms derived from genome editing technology. Specifically, organisms derived from genome editing are exempt from regulation if any of the following is applicable:

(i) The genetic modification is a change resulting from the cellular repair of a targeted DNA break in the absence of an externally provided repair template.

(ii) The genetic modification is a targeted single base pair substitution.

(iii) The genetic modification introduces a gene known to occur in the plant’s gene pool or makes changes in a targeted sequence corresponding to a known allele of such a gene or a known structural variation present in the gene pool.

(iv) The Administrator may propose to exempt plants with additional modifications, based on the outcomes of conventional breeding.

Both the revised regulations of the USDA and Japan’s Cartagena Act agree that the use of templates should be regulated. Additionally, the USDA regulations on genetically modified crops were triggered by the use of DNA fragments of microorganisms, typically *Agrobacterium*, which are plant pests. However, the recent revision of the Federal Regulations has shifted the focus of the regulation away from these methods to the plant pest risk posed by the crops themselves. The new regulations also allow development companies to make their own decisions, thereby reducing the burden on the development side and shifting the administrative focus to newly developed biotechnology products (NASEM 2017). However, developers can request confirmation from the USDA for products that are exempt from regulation. For more information of the CFR (Code of Federal Regulations), it is advisable to refer to the USDA-APHIS website.

### EPA regulatory policy

The EPA has regulatory authority over pesticide ingredients produced in plants or PIPs under the FIFRA. PIPs include pesticide components such as the *Bacillus thuringiensis* (Bt) toxin Cry1A, which is produced by Bt corn and other crops. Preregistration of these PIPs is mandatory, and tolerances in food and feed are set by the Federal Food, Drug, and Cosmetic Act (FFDCA).

At the end of August 2020, the EPA proposed to revise the FIFRA and expand the exemption provisions granted previously to plants derived from conventional breeding to include plants derived from new technologies such as genome editing. It also proposed to exempt PIPs from the tolerance levels set in the FFDCA. The final rule, published on May 25, 2023, divided PIPs into two categories:

First category: PIPs in which genetic engineering has been used to insert or modify a gene to match a gene found in a sexually compatible plant.

Second category: Loss-of-function PIPs in which the genetically engineered modification reduces or eliminates the activity of a gene; thus, contributing to the plant resistance to pests. Loss-of-function PIPs is a concept established for the first time in the regulatory revision and is a classification reflecting the characteristics of genome editing technology.

The first category requires developers to notify to the EPA, whereas developers can self-determine for the second category. Interestingly, the original proposal allowed for self-determination in all categories, but the final regulations by the EPA are more stringent than those of the USDA.

Although the EPA has taken action to require notification for certain types of products, the EPA’s revisions to its regulatory exemption for PIPs might be related to the Presidential Order issued on June 11, 2019 (Executive Order 13874, 84 Fed. Reg. 27899), i.e., “Modernizing the Regulatory Framework for Agricultural Biotechnology Products”, which substantially favors the exemption for low-risk products.

### FDA regulatory policy

With the “Statement of Policy: Foods Derived From New Plant Varieties” issued in 1992, the FDA has accepted voluntary consultations from companies regarding genetically modified foods. Although the consultation procedure has undergone several revisions since 1992, the basic framework remains unchanged. The safety of genetically modified foods is confirmed on a case-by-case basis. This voluntary consultation system provides a very flexible framework that can include new technologies such as genome editing as implied by the terms “new plant varieties”. While the consultation system is considered effective in the United States, the FDA collected public opinions related to genome editing technologies in January 2017. However, the FDA, which has not legislated yet even in the case of genetically modified foods, is not currently expected to introduce regulations for genome-edited products, as far as plants are concerned.

The FDA completed a voluntary consultation process in February 2019 regarding Calyxt’s Calyno oil, which derives from soybean with high oleic acid content and is a food product using genome editing technology. This oil is now on the market.

The FDA regulates genetically modified animals as well as genetically modified foods under the FFDCA. Regulations regarding genetically modified animals are handled by the Office of Veterinary Medicine (CVM) within the FDA. For animals derived from genome editing, the Draft Guidance for Industry on Intentional Modification of DNA in Animals (GFI #187) was published at the end of Barack Obama’s presidency (January 2017). Here, in contrast to the treatment of plant-derived products, animals modified with genome editing technology are under the same regulatory regime as genetically modified animals. The FDA policy has raised concerns from companies and developers, and the Department of Health and Human Services (the headquarter of the FDA) signed a memorandum of understanding (MOU) with the USDA in January 2021 to discuss the possibility of shifting jurisdiction over livestock animals from the FDA to the USDA. However, no major development has been reported yet (as of July 2023). In March 2022, the FDA decided “not to regulate” genome-edited short-hair cattle by exercising enforcement discretion. In the case of the genetically modified GloFish, the FDA also exercised enforcement discretion not to regulate on the grounds that there was no anticipated environmental impact. However, the exercise of such discretionary action is only taken after an application for approval has been filed, and there remains a lack of predictability on the part of the applicant. The FDA can take this kind of action on a case-by-case basis when there is little or no concern about a new product when an application is filed with the FDA.

## Regulations in Europe

### Regulations in the European Union

Since 2007, the European Union has been considering the regulation of products derived from new breeding techniques (NBTs) because the current GMO regulations are not adapted to these products. The European Commission established a New Techniques Working Group to examine the regulatory status of NBTs and other technologies that existed at the time. The technological trends and regulatory of eight new technologies, of which the regulatory status was unclear, were assessed at the European Joint Research Center (Lusser *et al.* 2011). As the results of these studies became clear, industry associations (e.g., Europabio and European Seed Association) were in favor of the technology not needing regulation, whereas the European Parliament and environmental groups (joint statement of environmental non-governmental organizations [NGOs] in March 2016) considered these products GMOs. Organic farming organizations (IFOAM EU Group) and others also expressed concerns about using seeds obtained using NBTs in organic farming.

A major policy development of the regulatory status of NBTs occurred in July 2018 with a ruling by the Court of Justice of the European Union (CJEU). Indeed, environmental groups sued the French government over the legal status of mutation breeding. Consequently, French courts asked the CJEU for a legal interpretation of using artificial mutation technologies, including genome editing technologies. In July 2018, the CJEU ruled on the legal status of products derived from artificial mutation technologies (mutagenesis) under the Environmental Release Directive (CJEU 2018). In short, organisms derived from mutagenesis are considered GMOs and are subjected to the legal obligations of the Directive. However, such organisms with a long history of safe use, for example, organisms derived from radiation breeding, were excluded from the scope of regulation in accordance with the provisions of Annex 1B of the Directive. In other words, organisms derived from genome editing technologies without a long history of safe use are GMOs and subjected to regulation under the European Union’s Environmental Release Directive. The impact of the 2018 ruling of the CJEU had repercussions outside the EU. For example, in the Republic of South Africa, the definition of GMOs was almost identical to that of the European Union’s Directive, and therefore, in accordance with the legal interpretation in the European Union, organisms subject to genome editing technology has been regulated as GMOs in South Africa since October 2021.

Subsequently, in February 2020, the French State Council announced that mutagenesis techniques, such as the use of radiation *in vitro*, should also be regulated. However, no regulation was implemented, partly due to opposition from the European Commission. Therefore, NGOs filed another lawsuit against the French government. The CJEU Grand Chamber ruled in February 2022 that any technology that (1) is used conventionally and (2) has a long history of safe use, whether *in vitro* or *in vivo*, is different from mutagenesis technologies such as genome editing technologies in the sense that it causes random mutations and that it is appropriate to exempt it from the GMO regulation. Cell fusion and induction of ploidy, which are *in vitro* techniques, were also originally exempted from the regulation, and they were judged to be in the same category as such treatments.

The CJEU’s ruling in 2018 has had a significant impact on the position of genome editing technology, and the European Commission instructed various EU agencies to gather information on new genomic techniques (NGTs), including genome editing technologies (e.g., regulatory status in member states, detection technologies, risk assessment, marketization trends, and ethical considerations). This information has been compiled by the European Commission and presented in April 2021. In September 2021, the European Commission introduced a roadmap to consider a legal framework for targeted mutagenesis and cisgenesis in plants, which was open for public comment.

Based on the above considerations, the European Commission proposed a new regulation in July 2023. The draft regulations are limited to crops using NGTs. Several conditions have been set, and if they are met, the crops derived from NGTs will be exempted from the regulation, including safety assessment, labeling, and traceability. The provisional conditions are specified in Annex I of the proposed rule. The conditions are as follows: (1) substitution or insertion of no more than 20 nucleotides; (2) deletion of any number of nucleotides; (3) the genetic modification does not interrupt an endogenous gene: (a) targeted insertion of a contiguous DNA sequence existing in the breeder’s gene pool; (b) targeted substitution of an endogenous DNA sequence with a contiguous DNA sequence existing in the breeder’s gene pool; (4) targeted inversion of a sequence of any number of nucleotides; (5) any other targeted modification of any size on the condition that the resulting DNA sequences already occur (possibly with modifications as accepted under points (1) and/or (2)) in a species from the breeders’ gene pool (European Commission 2023). Note that NGT-derived crops are banned from use in organic farming, and it is required to label NGT-based seeds as not to be used for organic farming.

### Regulations in the United Kingdom

After its separation from the European Union in February 2020, the United Kingdom has developed a new framework to replace policies based on previous EU regulations and directives, including those related to genetic modifications. In the United Kingdom, which has a leading industry in the life sciences, a new law on utilizing genome editing technology has been considered by the Parliament. Specifically, the Genetic Technology (Precision Breeding) Act 2023 was passed on March 23, 2023. With respect to plants and animals that meet certain requirements of precision breeding and food/feed derived from them, the Act allows for a relaxation of various regulations to which GMOs are subjected. Specific implementing regulations have been proposed by the Food Standard Agency in November 2023. The definition of “precision breeding” is provided in Article 1 (2) of the Act as follows:

(2) For the purposes of this Act, an organism is “precision bred” if—

(a) any feature of its genome results from the application of modern biotechnology, (b) every feature of its genome that results from the application of modern biotechnology is stable, (c) every feature of its genome that results from the application of modern biotechnology could have resulted from traditional processes, whether or not in conjunction with selection techniques, alone, and (d) its genome does not contain any feature that results from the application of any artificial modification technique other than modern biotechnology. As for the “traditional processes” mentioned above, various techniques are listed for plants and animals, such as sexual fertilization, spontaneous mutation, and *in vitro* fertilization. Please refer to Article 1 (6) of the Act.

The major points incorporated in the Precision Breeding Act are as follows based on the Explanatory Notes of the Genetic Technology (Precision Breeding) Bill.

(1) Two types of notification systems, one for marketing purposes and the other for non-marketing purposes, such as research and development, are stipulated.

(2) Animals subjected to precision breeding techniques are additionally subjected to health and animal welfare examinations and report on their status is mandatory.

(3) A public register is to be established for the information reported.

(4) A marketing authorization system has been introduced for food and feed derived from precision breeding technology, and it will be subject to a proportionate risk assessment.

(5) The Food Standards Agency (FSA) will be responsible for maintaining a public register of food and feedstuffs licensed for sale.

(6) Most of the Act will apply only in England and Wales.

The specific operation of this law will become clear once the implementing regulations have been clarified. In any case, the United Kingdom, as an independent country from the European Union, has progressed toward the active commercial use of genome editing technology.

## Regulations in Argentina and other South American countries

South American countries, especially Argentina, Brazil, Chile, Paraguay, and Colombia, have clarified their regulatory positions in favor of using genome editing technology, and commercialization has proceeded. In this subsection, only Argentina is mentioned as a representative case.

In May 2015, Argentina’s Ministry of Agriculture, Rural Development, and Fisheries (MAGyP) established a so-called “prior consultation procedure” for crops derived from NBTs such as genome editing (Decision 173/15). In 2021, the MAGyP clarified rules for prior consultation procedures, including applications to animals and microbes (Decision 21/2021). In prior consultation, new combination(s), i.e., foreign gene(s), are considered in terms of whether they have been introduced and remain in the genome. The Ministry accepts the prior consultation and then requests the Biosafety Committee (CONABIA), a governmental safety assessment body, to review the request and decide whether the product should be subjected to regulation (Entine *et al.* 2021, Whelan and Lema 2015).

Even if a crop is designated nonGMO, the Biosafety Committee may request the relevant government department (e.g., variety registration department) to consider additional measures if it finds that the crop is novel or has special characteristics. The final decision on permitting commercialization is made by these government departments. The Argentinean approach of making regulatory decisions based on the presence or absence of novel gene(s) has been adopted by neighboring countries, such as Brazil and Chile, and is becoming a common rule in neighboring trade blocs. The results of the preconsultation procedure are not public to avoid stigmatizing certain technologies from conventional breeding and discriminatory treatment.

The number of domestic developers of genome-edited products already notified to the Argentinean government has increased (from 8% to 59%) compared with that of developers of genetically modified crops (Whelan *et al.* 2020). Although specific applications have not been identified, Whelan *et al.* (2020) have mentioned oilseed crops, animals, horticultural crops, and grains. Most developers of genetically modified crops are multinational companies, but domestic companies are now commercializing genome editing technology, which should affect the domestic economy positively.

## Regulations in Oceania

### Regulations in Australia

In Australia, environmental safety of GMOs is regulated by the Office of the Gene Technology Regulator (OGTR 2018) under the Gene Technology Act 2000 and its implementing regulation, the Gene Technology Regulations 2001. Because NBTs such as genome editing technologies are emerging, the OGTR, after several years of investigations, notified a law on April 4, 2019, amending the current Gene Technology Regulation, the Gene Technology Amendment Regulations 2019. The revision clarifies that genome editing technologies involving site-directed nuclease 1 (SDN-1) are exempt from the current regulation, whereas genome editing technologies that use extracellular, artificially created templates (SDN-2) are subject to the regulation. With regard to SDN-2, it should be noted that the definition of SDN-2 differs from country to country (Jones *et al.* 2022). This paper does not organize the regulations of each country according to the SDN classification, and this is due to the fact that these understandings of SDN-2 are different.

The main revision made in 2019 are as follows: (1) revision of Appendix 1A (technology that is not a gene technology) clarifies the conditions under which RNA transfection is not considered a gene technology, (2) revision of Appendix 1 (organisms that are not GMOs) adds six items, including for specifying whether no template is used and matters related to null segregants, and (3) creation of Appendix 1B (technology that is gene technology) that specifies oligonucleotide-directed mutagenesis (ODM) and technologies that use templates. In addition, new regulations regarding gene drives were included because strict control of gene drives is required.

Although the revision excludes some genome editing technologies (SDN-1) from the scope of regulation, it does not fundamentally change the nature of the process-based regulation adopted by the OGTR so far. Additionally, because the revision regulates template-based technologies, it is similar to the USDA regulations and the policy under Japan’s Cartagena Act discussed thereafter. Another notable aspect of Australia’s revision process is that the revision of the system and the corresponding preparatory work were conducted very thoroughly. In Australia, the National Gene Technology Scheme is reviewed every five to six years (2006, 2011, and 2017), and various aspects of the system, including the adequacy of regulations and response to new technologies, are inspected. National opinion polls (e.g., Cormick and Mercer 2019) are also conducted to ascertain public attitudes. The decision to revise the regulations was based on a comprehensive review of these activities over the past years.

### Regulations in New Zealand

New Zealand has authorized very few GMOs so far, and no field trials are currently conducted. The country has very strict regulations regarding biodiversity conservation, including quarantine of invasive species.

In New Zealand, as in the European Union, organisms derived from genome editing are subject to GMO regulations. Like in the European Union, this decision was triggered by a court case. In February 2013, the Environmental Protection Authority (EPA) was in favor of regulating the development of trees using zinc-finger nuclease 1 (ZFN-1) and transcription activator-like effector nucleases (TALENs). However, in April 2013, a senior EPA official ruled that these genome-edited trees were not subjected to regulation under the country’s GMO rule, the Hazardous Substances and New Organisms Act (HSNO Act). An environmental NGO appealed against this administrative decision and filed a lawsuit. In May 2014, the High Court ruled in favor of the plaintiff NGOs’ arguments (New Zealand High Court 2014), finding that ZFN-1 and TALEN should be subjected to HSNO regulation. In the High Court judge’s written decision, these genome editing technologies were considered novel and not well established scientifically. Therefore, in view of the precautionary approach on which the HSNO Act relies, the judge ruled that it was not appropriate to exclude these techniques from the HSNO Act. Note that the EPA staff was originally was in favor of regulating the development of genome-edited trees (February 2013). However, a senior EPA official deemed it to not be regulated and made this EPA’s final decision (April 2013). At the conclusion of the court case, the EPA staff’s original decision was upheld.

In response to the ruling, the EPA revised the relevant statutes to explicitly state that products generated using mutagenesis techniques before July 29, 1998 (the effective date of the HSNO Regulation), would be treated as nonGMOs. This means that any product created using mutagenesis technologies (including genome editing technologies) developed after July 1998 is subjected to GMO regulations.

### Food Standards Australia and New Zealand (FSANZ) policy

In New Zealand, the EPA is responsible for environmental safety, but food regulations are under the jurisdiction of the FSANZ, which has been established jointly by Australia and New Zealand. In particular, the revision of Food Standards, which defines genetically modified foods, became an issue with the advent of genome editing technologies. Specifically, in February 2018, the FSANZ collected opinions on the revision, which were compiled in December 2019. A draft revision of the Food Standards (P1055) was published in October 2021. Until now, the FSANZ has defined genetically modified food as “food produced using gene technology” and has considered any food meeting this definition subjected to regulation. In the current proposal, genome editing technology is included in gene technology; however, the FSANZ has indicated that it will designate foods exempted from regulation based on the results of risk assessments and other factors. Exemptions include foods from which foreign genes have been removed, foods with characteristics that can be produced by conventional breeding, and processed foods that do not contain foreign genes or new proteins. Prior safety review of foods that do not meet these exemption criteria will be performed. A final decision is expected to be made after further consideration.

## Regulations in Asian countries

Since 2019, Asian countries have been actively revising regulations related to products derived from genome editing technology. Regulations in Japan, China, India, and the Philippines are discussed below as these countries have clarified their regulatory policies.

### Regulations in Japan

In Japan, the regulations surrounding genome editing technology have been mainly formulated by the Ministry of the Environment (which has jurisdiction over the Cartagena Act) and the Ministry of Health, Labour and Welfare (which has jurisdiction over the Food Sanitation Act). An overview is presented thereafter (Matsuo and Tachikawa 2022).

Based on the discussion at the Central Environmental Council, the Ministry of the Environment issued a Notice of the Director of the Nature Conservation Bureau, “Handling of organisms obtained using genome editing technologies and not regarded as ‘living modified organism’ specified in the Cartagena Act” (February 8, 2019). The notice states that organisms in which no foreign genes remain are exempted from regulation. Specifically, SDN-1 modifications are not regulated, whereas SDN-2-based techniques are regulated because SDN-2 uses a template (which corresponds to a “technology for processing nucleic acids outside the cell” regulated under the Cartagena Act). Technologies using SDN-3 are also regulated because SDN-3 introduces foreign gene(s).

Although products of SDN-1 are exempted from the regulation, the government requests developers to provide information before placing the product on the market. A summary of this information is published on the government’s website. The information to be provided covers eight items, including “changes in traits” and “consideration of the possibility of biodiversity impacts”. As more knowledge is acquired, revisions of the procedure and/or contents of information provision would be expected in the future.

In addition, the Ministry of Health, Labor and Welfare (MHLW) has decided on a policy for handling foods obtained through genome editing technology on September 19, 2019. Specifically, based on the review by the Pharmaceutical Affairs and Food Sanitation Council of the MHLW, “Food Hygiene Handling Procedures for Food and Additives Derived from Genome Editing Technology” (Decision by the Councillor for Environmental Health and Food Safety, Minister’s Secretariat) were issued. Rather than differentiating modifications obtained using SDN-1, SDN-2, or SDN-3, it has been decided that “risks that could occur even with conventional breeding techniques” are exempted from the regulation. Thus, SDN-1 modifications are not subjected to regulation, while SDN-2 is determined on a case-by-case basis (SDN-3 is subjected to regulation). As for SDN-2, the regulation of the MHLW thus differs from that of the Cartagena Act in Japan. The MHLW also requires notification of items that are not subjected to regulation based on prior consultations and publishes a summary on its website. The information to be submitted for prior consultation include confirmation that allergens are not produced, confirmation that the levels of toxic substances are not increased, and changes in nutritional components when metabolic systems are altered (Kondo and Taguchi 2022).

By the end of October 2023, six cases have been notified to the Ministry of the Environment and the MHLW. Those are tomatoes with high GABA accumulation (two bred lines), sea bream with increased edible portion, high-growth tiger puffer fish, waxy corn, and high-growth Olive flounder. The Ministry of Economy, Trade and Industry was also notified of one case involving the microorganism Euglena. All these cases, except the waxy corn, were developed by start-up companies in Japan.

### Regulations in China

In January 2022, the Chinese government (Ministry of Agriculture and Rural Affairs, MARA) published “Guidelines for Safety Evaluation of Agricultural Gene-Edited Plants (Trial)”. If the risk posed by a genome-edited plant is low, a small-scale intermediate test is conducted. The results of the test are submitted to the MARA to apply for a safety certificate for commercial production. If the anticipated risk is high, a larger scale test is required.

The above-mentioned guideline might aim to promote research and development and commercial use by maintaining China’s existing GMO regulations. In other words, it might be a policy for regulating organisms derived from genome editing technology using existing laws, while introducing a simplified procedure (i.e., one can apply for a safety certificate after a small-scale test). In recent years, China has also been actively revising its regulations on genetically modified crops, and there are indications that China is trying to promote a smoother use of life sciences. The first safety certificate (valid for five years) was issued by the new regulation system in April 2023 for high oleic soybeans, which are developed by a private company in Shandong Province.

### Regulation in India

On March 30, 2022, the Ministry of Environment, Forests and Climate Change of India issued an Office Memorandum (MOEFCC 2022), which states that plants produced using SDN-1 and SDN-2 and that do not contain foreign gene(s) are exempted from GMO regulations. In other words, notification was given that Articles 7 to 11 (import/export, manufacturing and processing, environmental releases, food use, etc.) of the GMO regulations are not applicable in those cases.

In addition, the Department of Biotechnology, Ministry of Science and Technology issued “Guidelines for Risk Assessment of Genome-Edited Plants” in May 2022, and “Guidelines for Regulatory Review of Standard Operating Procedures” were published in October 2022 to specify application procedures, including where the applicant should submit the application and the information to be submitted to the government when notification is required.

### Regulations in the Philippines

The Philippines has been conducting technical and regulatory studies on plant NBTs since 2016. In particular, in June 2019, a decision has been made to develop a government policy and guidelines on plant breeding innovations under the leadership of the Department of Agriculture. Based on the above considerations, the Department of Agriculture issued Memorandum Circular No. 8 in May 2022 and published rules and procedures for the marketing of products based on plant breeding innovations. In the new rules, products that do not contain foreign gene(s) (new combinations of genetic material) are exempted from regulation. Specifically, developers have to provide information to the Plant Industry Bureau of the Ministry of Agriculture and follow specific procedures. If the product is exempt from the GMO regulation, a certificate is issued to the developer and the information, excluding confidential business information, is published on the government website.

## Conclusion

Here, we have provided an overview of regulations of genome editing technologies in various countries. The specific regulations differ from country to country, resulting in a patchwork of regulations. However, regulations are converging (Tachikawa and Matsuo 2023) as an increasing number of countries are adopting one of two policies. Some countries, such as Argentina, Japan, India, and the Philippines, are introducing rules that require notification and confirmation from the government even if the product is not considered a GMO. Other countries, including China, the United Kingdom, and possibly FSANZ, are introducing rules with slightly less restrictive regulations than those for regular GMOs even when the product is considered a GMO. Only a few countries consider products obtained using NBTs as fully equivalent to GMOs (the European Union and New Zealand, although the European Union is currently preparing a new regulation) or, in contrast, to conventional breeding (USDA in the United States or OGTR in Australia), in which case there is no need for notification. Many other countries are expected to consider new regulations in the future. It will be necessary to monitor regulatory trends and their implications closely.

Note: This paper is based on the following paper with necessary corrections and updates.

Tachikawa and Matsuo (2023) “Regulatory and acceptance status of genome editing technologies in Japan and abroad” in *Application of Genome Editing Technology in Pharmaceuticals, Agriculture, Water and Livestock, and Biotechnology Fields and Countermeasures for Challenges*. Gijutsu Joho Kyokai (Tokyo, Japan).

## Author Contribution Statement

MT and MM discussed the study design and conducted research reviews and interviews. MT drafted the manuscript based on the study and MM edited the manuscript. All authors contributed to the article and approved the submitted version.

## Figures and Tables

**Table 1.** Classification of regulatory oversight of genome edited products in various countries

How the product is treated under the regulation: GMO/non-GMO	Applied regulatory oversight	Country or authority
GMO	GMO regulation as it is	NZ (EPA), South Africa
GMO	Simplified GMO regulations	UK, FSANZ*, China
non-GMO	Exempted but with confirmation by regulatory authority	EU*, Argentina and South America, Japan, India, Philippines
non-GMO	Confirmation not required by regulatory authority	US (USDA), Australia (OGTR)

Note: An asterisk (*) indicates that it is under consideration. Since products using SDN-3 is subject to GMO regulations, SDN-3 is excluded from this Table. Adapted from Tachikawa and Matsuo (2023).
